# Editorial: Hypoxia and exercise: Tissue specific and systemic adaptive responses

**DOI:** 10.3389/fphys.2022.1095416

**Published:** 2022-11-24

**Authors:** Olivier Girard, Rui Duan, Katsuhiko Suzuki, Xu Yan

**Affiliations:** ^1^ School of Human Sciences (Exercise and Sport Science), The University of Western Australia, Perth, WA, Australia; ^2^ School of Physical Education and Sports Science, South China Normal University, Guangzhou, China; ^3^ Faculty of Sport Sciences, Waseda University, Tokorozawa, Japan; ^4^ Institute for Health and Sport, Victoria University, Melbourne, VIC, Australia; ^5^ Australian Institute for Musculoskeletal Sciences, Melbourne, VIC, Australia; ^6^ Department of Medicine—Western Health, The University of Melbourne, Melbourne, VIC, Australia

**Keywords:** exercice, hypoxia, systemic adaptations, tissue-specific adaptation, simulated altitude

## Introduction

Hypoxia exposure leads to lower oxygen availability. Animals and humans dispose of acute and long-term coping mechanisms to protect themselves from hypoxia. In fact, if individual adaptive capacities are insufficient or the environmental stimulus is too severe, hypoxia exposure may become detrimental for many organ systems notably exercising skeletal muscles and the brain. Conversely, positive physiological adaptations not only acutely enhance tolerance to hypoxia but can also induce sustained performance and health benefits. Our intention for this Research Topic was to invite submissions discussing the tissue specific and multi-systemic adaptations to hypoxia, and the combination of hypoxia and exercise. This Research Topic contains a series of eleven articles (i.e., two systematic reviews, two reviews, and seven original articles). This collection of articles provides new knowledge, and most importantly, an integrative view of some of the systemic and molecular mechanisms likely driving any hypoxia-induced adaptation or maladaptation.

### Acute and chronic exposure to terrestrial altitude

Acute hypoxia refers to a short exposure when rapid physiological responses occur in order to counterbalance the decrease in oxygen pressure and delivery at the different stages of the oxygen cascade (from alveolar to mitochondria). Zhang and Wang first demonstrated that pharmacokinetic changes of sildenafil were mainly caused by the decrease in protein expression of CYP3A4 enzyme in rats acutely exposed to 4,300 m, but not 2,300 m, above sea level. Authors argued that a multi-factor regulation mechanism likely dictates changes of the substrate sildenafil pharmacokinetic process, which seems closely related to the adjustments in blood gas, biochemical indicators and metabolic enzymes. Spectral-domain optical coherence tomography was then used by Yin et al. to quantify changes in the retinal structure in 109 healthy individuals after ascent to 3,700 m above sea level. Results showed that the ratios of mean thickness, inferior area, and nasal area were correlated positively with high-altitude headache. This provides new insights into the pathophysiology of high-altitude retinopathy (i.e., papilledema). Unlike acute exposure, chronic continuous hypoxia relies on prolonged exposure or permanent life in altitude with longer-term physiological adaptations. In their cross-sectional study including 475 children and adolescents living at 1,000 or 2,600 m above sea level, Mancero-Soto et al. concluded that the associated effects of endurance training on haemoglobin mass and blood volume were only observed after the onset of puberty. Additionally, authors stated that the large differences in haemoglobin mass and blood volume in adulthood between elite athletes and untrained individuals likely have genetic origins (yet of unknown origin). Overall, the outcome of hypoxia exposure and efficiency of adaptations are largely determined by individual predispositions and vulnerabilities as well as by the “hypoxic dose” (i.e., severity, duration, and frequency of the stimuli).

### Intermittent hypoxia exposure

Intermittent hypoxic exposure corresponds to the repetition of hypoxic/normoxic cycles, while its effects range from deleterious (e.g., sleep-disordered breathing) to beneficial (e.g., hypoxia conditioning). The usefulness of intermittent hypoxia exposure (inspired oxygen fraction or FiO_2_ = 14.5%) to prevent intense exercise training-induced reductions in haemoglobin concentration was assessed in animal and human studies. Firstly, Weng et al. exposed six-week-old male Sprague-Dawley rats to progressive intense treadmill exercise training over 3 weeks followed by 3 weeks of training with intermittent hypoxia exposure (either for 1, 2, or 1 h + 1 h separated by a 3-h interval after the exercise sessions). Authors concluded that all these intermittent hypoxia exposure strategies (i.e., no difference between treatments) could be used to increase renal erythropoietin and alleviate intense exercise training-induced reductions in haemoglobin concentration. Secondly, Weng et al. reported that 1 h of normobaric hypoxia exposure five times a week over 4 weeks was sufficient to partially restore the low haemoglobin concentration in trained swimmers, but also blunt the decrease in red blood cells and haematocrit. Overall, humans might be more sensitive to the intermittent hypoxia exposure intervention than rats.

### Responses to hypoxia and exercise stressors when combined

Ambient hypoxia exposure and exercise, or a combination of both stressors, likely influence the cerebrovascular and muscle regulation interplay. To illustrate, pacing strategy during a 250-kJ cycling time-trial was impaired more after 24 h of hypobaric (3,450 m above sea level) than normobaric (FiO_2_ = 13.6%) hypoxia, which may relate to altered cerebrovascular responses (Rupp et al.). By aiming to maintain an equivalent oxygen delivery to the brain, individuals at terrestrial altitude likely adopted a more “protective” strategy, in conjunction with greater impairments in cerebral blood flow and prefrontal motor cortex oxygenation, leading to lower overall cycling performance compared to simulated altitude. Oxygen deprived conditions can also influence exercise-related cardio-vascular system adjustments. A brief exercise bout of mild intensity (30% of maximal aerobic power) in acute normobaric hypoxia (FiO_2_ = 13.5%) did not impair systolic or diastolic functions during the ensuing recovery period as evaluated from echocardiographic, Doppler, and tissue Doppler measures (Magnani et al.). Rather, stroke volume was well preserved and systolic and early diastolic functions were actually enhanced by exercise in hypoxia. Finally, the review by Lemieux and Birot on angio-adaptative responses to hypoxia summarizes the remarkable yet complex molecular plasticity of the capillary microvasculature (i.e., capillary-to-myofiber interface).

### Therapeutic use of hypoxia

In recent years, the possibility of using hypoxic exposure as a novel therapeutic strategy (i.e., hypoxia conditioning) to improve health outcomes has gained popularity. In their review of the impact of high-altitude hypoxia on bone defect repair, Chen et al. discussed the possible mechanisms related to ion channels, reactive oxygen species production, mitochondrial function, autophagy, and epigenetics. While there is currently no clear optimal treatment plan for bone defects at high altitudes, this review also provides a foundation for future targeted, personalized, and precise bone regeneration therapies. Another systematic review with meta-analysis including 19 studies (a total of 444 participants) showed that the effects of exercise training in hypoxia and normoxia on fat loss in overweight and obese adults are not different (Chen et al.). Subgroup analysis of different age of participants, hypoxia dose, exercise frequency, and duration failed to demonstrate hypoxia-related effects on body composition, glycometabolism, and lipometabolism. Although hypoxia conditioning has potential in enabling a healthier lifestyle and reduction of related risk factors in cardio-metabolic diseases, more work needs to be done to identify the most effective strategies that are also safe and well tolerated.

### Moving forward

A vast number of potential health- and performance-promoting hypoxia applications exist. The strong sense one gets from reading the Hohenauer et al. systematic review is that a positive and small tendency can be seen over the past 40 years for the increase in the methodological quality of clinical trials examining hypoxia-related physiological responses. To accelerate this trend, authors recommended that future studies should incorporate adequate blinding procedures (if possible), concealed allocation, and baseline comparability. By adhering to these principles, well-calibrated hypoxic interventions could be developed and refined to maximize health and performance outcomes.

